# Chromatin structure can introduce systematic biases in genome-wide analyses of *Plasmodium falciparum*

**DOI:** 10.12688/openreseurope.14836.1

**Published:** 2022-06-10

**Authors:** Sebastian Baumgarten, Jessica Bryant

**Affiliations:** 1 Plasmodium RNA Biology Group, Pasteur Institute, Paris, Paris, 75015, France; 2 Biology of Host-Parasite Interactions Unit, Pasteur Institute, Paris, Paris, 75015, France; 3 CNRS ERL9195, Paris, 75015, France; 4 INSERM U1201, Paris, France

**Keywords:** Chromatin structure, ChIP-seq, Plasmodium falciparum, read alignment

## Abstract

**Background: **The maintenance, regulation, and dynamics of heterochromatin in the human malaria parasite, *Plasmodium falciparum,* has drawn increasing attention due to its regulatory role in mutually exclusive virulence gene expression and the silencing of key developmental regulators. The advent of genome-wide analyses such as chromatin-immunoprecipitation followed by sequencing (ChIP-seq) has been instrumental in understanding chromatin composition; however, even in model organisms, ChIP-seq experiments are susceptible to intrinsic experimental biases arising from underlying chromatin structure.

**Methods:** We performed a control ChIP-seq experiment, re-analyzed previously published ChIP-seq datasets and compared different analysis approaches to characterize biases of genome-wide analyses in *P. falciparum*.

**Results: **We found that heterochromatic regions in input control samples used for ChIP-seq normalization are systematically underrepresented in regard to sequencing coverage across the *P. falciparum* genome. This underrepresentation, in combination with a non-specific or inefficient immunoprecipitation, can lead to the identification of false enrichment and peaks across these regions. We observed that such biases can also be seen at background levels in specific and efficient ChIP-seq experiments. We further report on how different read mapping approaches can also skew sequencing coverage within highly similar subtelomeric regions and virulence gene families. To ameliorate these issues, we discuss orthogonal methods that can be used to characterize *bona fide *chromatin-associated proteins.

**Conclusions: **Our results highlight the impact of chromatin structure on genome-wide analyses in the parasite and the need for caution when characterizing chromatin-associated proteins and features.

## Plain language summary

Chromatin immunoprecipitation followed by sequencing (ChIP-seq) is the method of choice to identify where a chromatin feature associates with the genome on a global scale. However, the secondary structure and inherent sequence of the genome can be challenging for ChIP-seq analysis. Variation in DNA accessibility, similarity of multiple sequences, and DNA sequence diversity can lead to inaccurate analyses. In this study, we describe how these factors influence the analysis of genome-wide ChIP-seq data generated by Next Generation sequencing in the human malaria parasite *Plasmodium falciparum*. In particular, we observed that DNA regions associated with compact chromatin are underrepresented in samples used for ChIP normalization, leading to the identification of false enrichments across these regions. Furthermore, we show how the choice of options during the mapping of sequencing reads to the parasite genome and subsequent filtering steps can differentially affect regions with varying levels of similarity and nucleotide diversity. Together, these data highlight the sensitivity of genome-wide analyses to intrinsic chromatin features in the human malaria parasite and how orthogonal methods can be used to characterize chromatin-associated features.

## Introduction

Epigenetic regulation of transcription has become a major focus in the study of *Plasmodium falciparum*, the eukaryotic parasite that causes the most severe form of human malaria. An ever-increasing number of studies have attempted to elucidate how this parasite, which has relatively few specific transcription factors compared to other eukaryotes, maintains sophisticated programs of gene regulation throughout its complex life cycle. Accordingly, chromatin-immunoprecipitation followed by sequencing (ChIP-seq) has become widely used to identify the genome-wide enrichment of putative regulatory proteins. In *P. falciparum*, ChIP-seq has been instrumental in characterizing the dynamics of histone post-translational modifications, transcription factors, and other chromatin-associated proteins involved in transcriptional activation and silencing (reviewed in 1, 2). Because of its importance to parasite pathogenicity and transmission, the formation, maintenance, and dynamics of heterochromatinization uniquely of subtelomeric regions and individual central chromosomal clusters has received special attention. Heterochromatin silences genes such as the virulence gene families encoding variant surface antigens ( *e.g. var, rifin* and *stevor*) and *ap2-g,* a transcription factor that is essential for the differentiation of the parasite into the human-to-mosquito transmission stage.

**Figure 1.**
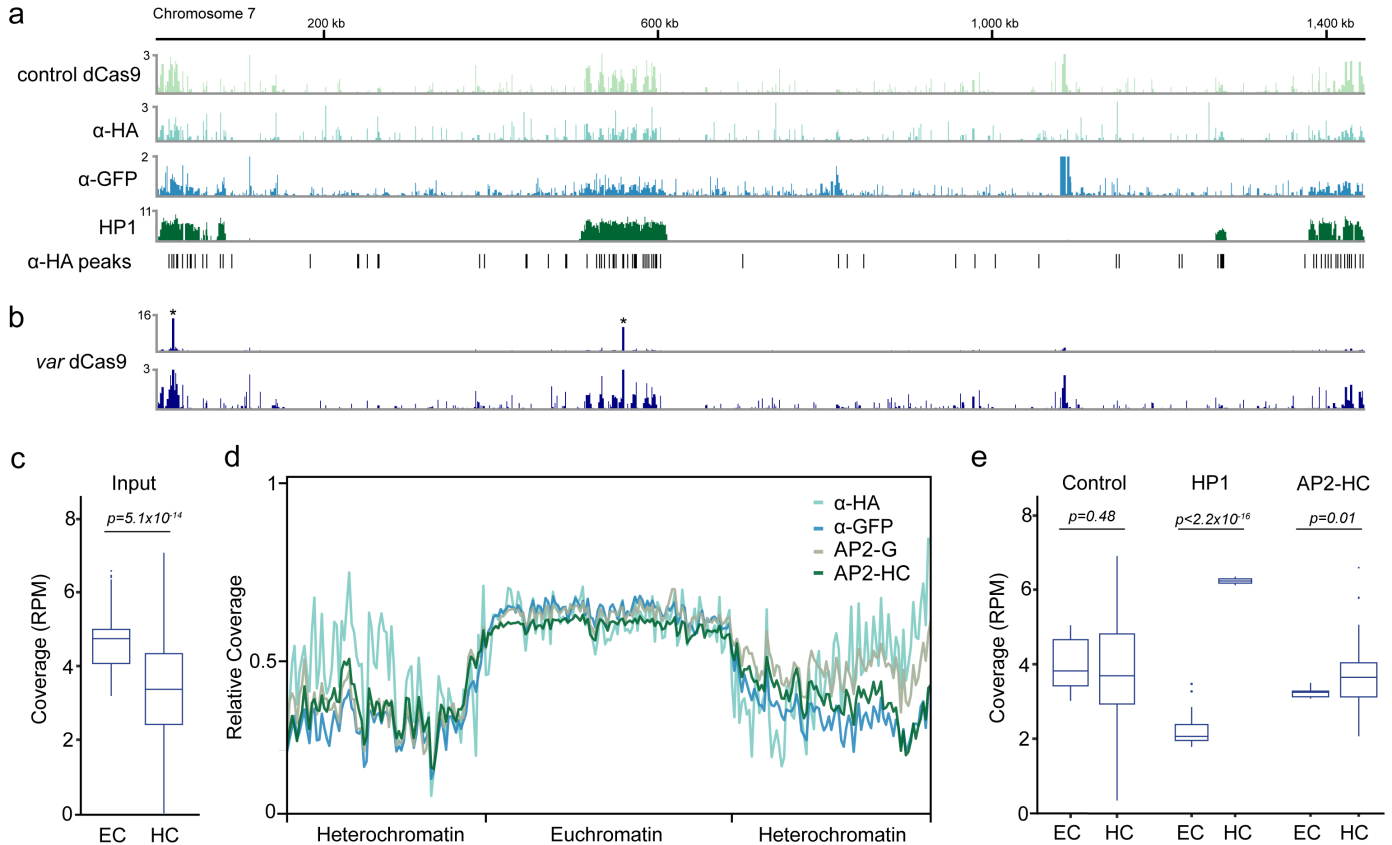
Enrichment of heterochromatic regions in control chromatin immunoprecipitation sequencing (ChIP-seq) experiments. **a**) Fold-enrichment (ChIP/input) of three control ChIP-seq experiments (control dCas9, α-HA, α-GFP) and the heterochromatin marker HP1 on chromosome 7. Significant peaks identified in the α-HA ChIP-seq experiment are indicated on the bottom. **b**) Fold-enrichment (ChIP/input) of a dCas9 ChIP-seq experiment on chromosome 7 scaled to the two peaks of intended dCas9 target sites (top, indicated with asterisks) and with decreased enrichment range (y-axis, bottom). **c**) Genome coverage (reads per million, RPM) in euchromatic (EC) and heterochromatic (HC) regions of ChIP-seq input libraries. **d**) Metagene plot of the 14 nuclear chromosomes of *P. falciparum* of ChIP-seq input libraries used for normalization. **e**) p < 2.2x10 ^-16^ Genome coverage (reads per million, RPM) in euchromatic (EC) and heterochromatic (HC) regions for three control ChIP-seq immunoprecipitations libraries (control dCas9, α-GFP, α-HA, ‘control’, left), HP1 (middle) and the heterochromatin-associated transcription factor AP2-HC (right).

 As the field of epigenetics progresses, experimental standards for genome-wide studies of chromatin have been set forth by the ENCODE project, including using appropriate controls, replicates, normalization, and read depth; however, there are *P. falciparum*-specific issues that have come to our attention in recent years that mostly concern heterochromatic or highly homologous regions of the genome. In a recent report where we characterized the *var* gene interactome with enChIP, we used an epitope-tagged enzymatically inactive Cas9 (‘dead’, dCas9) that was co-expressed with a non-specific guide RNA as negative control ^3^. Although the dCas9 protein had no intended target within the parasite genome, ChIP-seq analysis of this supposedly non-specific dCas9 showed a specific enrichment in heterochromatic regions across the parasite genome ( Figure 1a, top). As this result was unexpected, we sought to discover an explanation in order to optimize our protocol. Performing and re-analyzing additional control experiments and using previously published ChIP-seq experiments of *bona fide* chromatin-associated proteins, we found that heterochromatic regions across the parasite genome are systematically under-represented in input samples used for ChIP-seq normalization. In combination with non-specific or inefficient immunoprecipitations, this bias can lead to the identification of enrichment in heterochromatic regions. Here, we highlight specific pitfalls and intrinsic confounding factors associated with genome-wide analyses and how orthogonal methods can be used in the characterization of chromatin-associated proteins in the human malaria parasite.

## Results

In order to confirm the initial observation of background heterochromatin enrichment in the dCas9 control strain, we performed ChIP-seq on wild-type *P. falciparum* (3D7) ring stage parasites (12 hours post infection) using an α-HA antibody. In addition, using an identical pipeline (see Methods), we re-analyzed a control experiment that used an α-GFP antibody ^4^ as well as an α-HP1 ChIP-seq as positive control of a heterochromatin-associated protein ^5^. For all three control experiments ( *i.e.* control dCas9, α-HA and α-GFP), we observed similar enrichments across the subtelomeric and central heterochromatin clusters resembling the profile of HP1 ( Figure 1a). Peak-calling analysis on the α-HA experiment using macs2 identified 1,332 significant (q-value ≤ 0.05) peaks that were significantly overrepresented within heterochromatic regions (505 peaks, χ ^2^-test *p* < 2.2×10 ^-16^, Figure 1a). We next asked whether such an intrinsic bias could also be observed within a ChIP-seq experiment of a specific DNA-binding protein. We re-analyzed our data of a ChIP-seq experiment that used a dCas9-HA protein with a guide RNA specific to the upstream region of 17 *var* genes ^3^. Enrichment of the dCas9 protein was highly specific and robust at the targeted binding sites ( Figure 1b, top and 3); however, when we visually decreased the enrichment range (y-axis), we could also observe a background enrichment across heterochromatic regions similar to the enrichment observed for the control ChIP-seq experiments ( Figure 1b, bottom).

Because immunoprecipitated chromatin is normalized to corresponding input chromatin, the observed heterochromatic enrichment in control/non-specific ChIP-seq experiments could arise either from 1) an overrepresentation of heterochromatinized regions in the immunoprecipitation or 2) an underrepresentation of heterochromatinized regions in the input sample. When we compared input samples from six different ChIP-seq experiments ^3– 6^, we found that heterochromatic regions had significantly lower sequencing read coverage than euchromatic regions ( *p* = 5.1×10 ^-14^, Figure 1c). This was particularly true for heterochromatic subtelomeric regions, where input samples from ChIP-seq experiments performed by four different laboratories all showed significantly lower coverage than the euchromatic, central chromosomal regions ( Figure 1d) ^3– 6^. This underrepresentation of heterochromatic regions was not significant in the three control/non-specific immunoprecipitation samples *(p* = 0.48, Figure 1e). In contrast, immunoprecipitation samples for HP1 and a recently characterized heterochromatin-associated ApiAP2 transcription factor, AP2-HC, showed significantly higher read coverage in heterochromatic than in euchromatic regions ( *p* < 2.2×10 ^-16^ and *p* = 0.01, respectively, Figure 1e).

In addition to the issue of heterochromatin representation, the composition of the *P. falciparum* genome also poses a challenge for ChIP-seq analyses. First, the overall GC content of the *P. falciparum* genome is approximately 19%, the lowest reported for any genome known to date ^7^. Intergenic regions in particular show very low levels of nucleotide diversity and an average AT content of up to 90%. Second, subtelomeric regions and other regions containing members of multigene families share a high level of sequence similarity ^7, 8^ and show an elevated rate of recombination events ^9, 10^. Both low nucleotide diversity and high sequence similarity among multiple gene loci or genomic regions complicate the read mapping step of genome-wide analyses, since the true location of a sequenced molecule originating from such a gene/region cannot be exactly inferred.

With default settings, the popular short-read aligner bowtie2 only reports one alignment per read, and the confidence of the alignment corresponding to the true origin of the sequenced molecule is given by the mapping quality (MAPQ). The higher the mapping quality, the larger the difference between the best and the second-best possible alignment of a given read. MAPQs are reported as Q= -10*log _10_(p), where *p* equals the probability that the reported alignment is not the true location from where the sequenced molecule originated ^11^. Thus, a MAPQ value of 10 equals the probability of the reported alignment location being incorrect is 1/10, while a MAPQ value of 40 would indicate a probability of 1/10,000.

**Figure 2.**
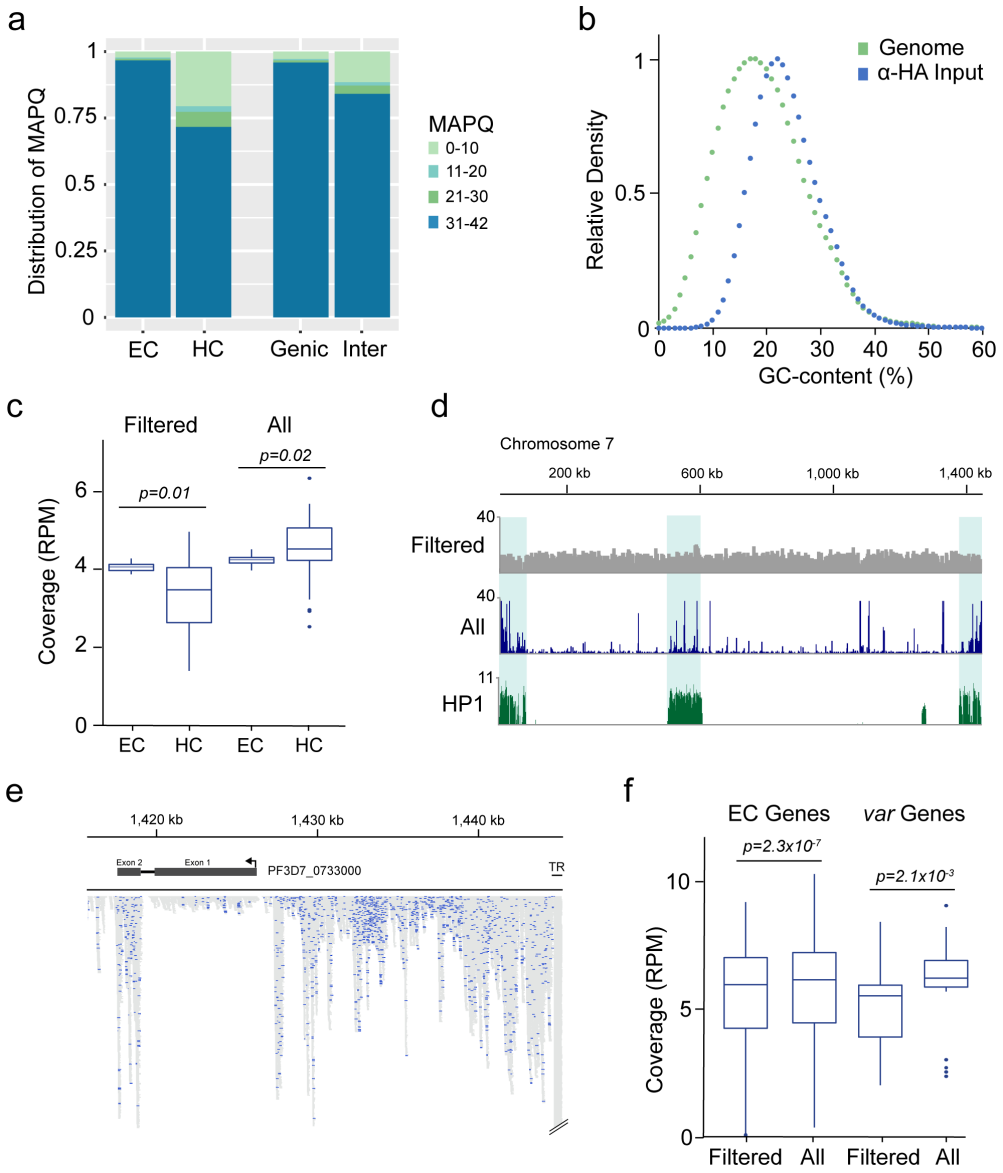
Read mapping biases between euchromatic and heterochromatic regions. **a**) Distribution of read alignment mapping quality (MAPQ) between euchromatic (EC), heterochromatic (HC), genic and intergenic (Inter) regions. **b**) Relative abundance of 100 bp windows sorted by GC content within the *P. falciparum* genome (Genome) and relative abundance of read alignments from the α-HA input library within 100 bp windows sorted by GC content (α-HA). **c**) Comparison of genome coverage (reads per million, RPM) within euchromatic (EC) and heterochromatic (HC) regions calculated from MAPQ-filtered alignments ( *i.e.*, reporting one alignment per read with MAPQ ≥ 30, ‘Filtered’) and from all possible alignments ( *i.e.*, reporting all alignments of a read mapping to multiple locations and without MAPQ-filtering, ‘All’). **d**) Genome coverage of MAPQ-filtered (Filtered) and all possible (All) alignments across chromosome 7. Fold-enrichment (ChIP/input) of the heterochromatin marker HP1 is shown on the bottom. **e**) Detailed view of read alignments at the end of chromosome 7 when all possible alignments per read are reported. Blue: Alignments with a MAPQ ≥ 30. Grey: Alignments of reads mapping to multiple regions in the genome. TR: Telomere repeat. Arrow indicates direction of transcription **f**) Comparison of mapping approaches as in **c**) for genes located in euchromatic regions (EC Genes) and members of the *var* multigene family ( *var* Genes). Coverage was calculated from MAPQ-filtered alignments ( *i.e.*, reporting one alignment per read with MAPQ ≥ 30, ‘Filtered’) and from all possible alignments ( *i.e.*, reporting all alignments of a read mapping to multiple locations and without MAPQ-filtering, ‘All’)

We used the input sample of the α-HA control ChIP experiment to compare the distribution of MAPQ values between alignments in euchromatic and heterochromatic regions. We found that 97% of aligned reads in euchromatic regions, but only 71% of aligned reads in heterochromatic regions had a MAPQ ≥ 31 ( Figure 2a). Even more striking, the percentage of all read alignments with a MAPQ ≤ 10 was 2% for euchromatic regions and 22% for heterochromatic regions ( Figure 2a). We observed a similar trend in the comparison of genic and intergenic regions, with 3% and 12% of all alignments within genic and intergenic regions, respectively, with a MAPQ ≤ 10 ( Figure 2a, right). Filtering for reads with high MAPQ values ( *i.e.* ≥ 30) therefore affects genic and intergenic regions disproportionally: when calculating the distribution of 100 basepair bins with a given GC content across the parasite genome, the peak of GC bins was at 18% ( Figure 2b). In contrast, the alignments from the α-HA input sample described above were biased towards higher GC content, with the peak of the distribution falling within the 22% GC bin. These data suggest that experiments aiming to characterize features potentially binding within these regions ( *e.g.* transcription factors) might require deeper sequencing to compensate for lower numbers of high-confidence alignments.

The discrepancy between different regions of the genome regarding the confidence of a read’s true origin is exacerbated when instead of only one alignment, every possible alignment of a read is reported during read mapping and no further downstream quality filter step is included. Using the α-HA input sample, reporting of only one alignment per read (default in bowtie2) and filtering for MAPQ ≥ 30 resulted in the underrepresentation of heterochromatic regions relative to euchromatic regions as described above ( *p* = 0.01, Figure 2c, left). Reporting all alignments for a given read (bowtie2 option -a) led to an overall increase in genome coverage for both euchromatic and heterochromatic regions. However, this increase in coverage was substantially higher for heterochromatic regions, leading to a significant overrepresentation of these regions compared to euchromatic regions ( *p* = 0.02, Figure 2c, right; Figure 2d). On average, we found almost twice the number of alignments within heterochromatic compared to euchromatic regions when all possible alignments were reported, although heterochromatin in our analysis made up only 12% of the total genome size. Within subtelomeres, loci sharing the highest sequence similarity showed the most pronounced increase in coverage, including the conserved exon 2 of members of the *var* multigene family, and especially the telomeric repeat regions ( Figure 2e). When multiple alignments of a read were reported, the average increase in coverage for genes located in euchromatic regions was only ~4 %, but ~30% for members of the *var* multigene family ( Figure 2f). These data demonstrate the drawbacks of studying an organism with multigene families showing high sequence similarity and the importance of using stringent mapping parameters for analysis of Next Generation sequencing data.

## Discussion

Challenges with ChIP-seq analysis surrounding heterochromatic regions and regions with similar sequences in the genome are not unique to *P. falciparum*; however, the presence of several multigene families in heterochromatic regions of the genome and low sequence diversity in intergenic regions makes *P. falciparum* a particularly difficult organism for generating robust ChIP-seq datasets. Chromatin fragmentation is a key step in any ChIP-seq protocol and can differ between samples or experiments, making it important to perform multiple replicates and to use an input sample from the same chromatin preparation used for the ChIP (see ENCODE ChIP-seq guidelines ^12^). Chromatin structure affects chromatin fragmentation whether sonication or enzymatic cleavage are used, and heterochromatin tends to be more difficult to fragment by sonication than euchromatin ^13^. Thus, fragments of the genome from heterochromatic regions tend to be longer than those from euchromatic regions and are not sequenced as efficiently and/or are lost when the sonicated chromatin is cleared by centrifugation. The underrepresentation of heterochromatic regions in the analyzed *P. falciparum* ChIP-seq input samples was not seen in the immunoprecipitated DNA from non-specifically binding features or control antibodies, which leads to spurious “enrichment” of heterochromatic regions when immunoprecipitated DNA is normalized to corresponding input DNA ( Figure 1). In contrast, for true heterochromatin-associated proteins ( *i.e.*, AP2-HC and HP1), we found that heterochromatic DNA was enriched compared to euchromatic DNA in the immunoprecipitated DNA sample ( Figure 1e). Moreover, in the case of specific and/or effectively immunoprecipitated chromatin features, background enrichment of heterochromatic regions is negligible compared to the real ChIP signal ( Figure 1b).

Another issue with analyzing ChIP-seq data in *P. falciparum* is the mapping of genomic regions with high sequence similarity and/or low nucleotide diversity. Allowing a read to map to multiple loci in the genome and reporting each alignment can result in false over-representation within multigene families or ribosomal genes, which show relatively high levels of homology. Similar issues are prominent and have been reported previously in model systems, for example regarding the repeat targets of PIWI-interacting RNA ^14^. To ensure robust and replicable data, analysis of ChIP-seq experiments should include stringent mapping and filtering steps for both input and immunoprecipitation samples and include 1) the reporting of only one alignment per sequenced read with a high alignment score, 2) the removal of PCR duplicates, and 3) filtering out alignments that might not represent the true origin of a sequenced read ( *e.g.*, low MAPQ in bowtie2). Performing Next Generation sequencing with longer and/or paired-end reads can help in assigning the origin of a sequenced read more accurately, especially with regard to heterochromatic genomic loci with high sequence similarity.

In addition to a rigorous analysis pipeline, experimental controls can provide additional confidence in a ChIP-seq data set, especially when performing ChIP-seq for the first time with an uncharacterized chromatin feature or antibody. First, a chromatin feature of interest should be present at high enough levels at the time point in the life cycle being investigated to result in a robust immunoprecipitation. Here, a basic immunoprecipitation followed by Western blot from a crosslinked sample should be used to determine whether the feature of interest can be enriched from the nuclear or chromatin fraction. For an epitope-tagged chromatin feature, a key control ChIP-seq experiment would be to use the antibody against the epitope tag with the same amount of input chromatin, but from the wild-type parent strain that does not contain any epitope-tagged proteins. For an uncharacterized antibody, Western blots and immunoprecipitation followed by mass spectrometry could provide evidence for specificity (see ENCODE guidelines), and immunoprecipitation with immunoglobulin G (IgG) could serve as a ChIP-seq control. Additional methods for validating ChIP-seq data are ChIP followed by quantitative PCR using highly specific primers (if possible).

If a region of the genome is enriched in both a test and control ChIP-seq experiment, it is still possible that the enrichment is real. To provide additional experimental support for true ChIP-seq enrichment of a chromatin feature, different orthogonal methods have been successfully used in *P. falciparum*. One option is to determine if the binding profile of the chromatin feature of interest is dynamic ( *e.g.*, enrichment may change depending on the life cycle stage or growth conditions of the parasite), as in 6. Another option is to detect changes in enrichment when the chromatin feature of interest or auxiliary factor is depleted ( *e.g.*, with a knockout or knockdown), as in 5. Even further support could be provided if the knockout/down affects the transcription of genes that are enriched for the chromatin feature in the ChIP-seq data, as in 3, 6, 15. One important caveat of this last type of analysis is that heterochromatinized multigene families expressed in a mutually exclusive manner will always appear to be differentially expressed if two different clones are used for the comparison. Thus, using an inducible knockdown/out system in the same parasite clone has to be used to obtain interpretable data for differential expression analysis of multigene families.

Using these bioinformatic and experimental techniques will allow the field of chromatin and epigenetics in *P. falciparum* to progress and reveal important processes in the gene regulation of this parasite. Maintaining high experimental and bioinformatic standards in the analysis of genome-wide features will ensure standing in the field of epigenetics and chromatin, which is so often dominated by model systems.

## Methods

### Chromatin immunoprecipitation

The ChIP-seq experiment for the α-HA samples was performed as described in detail in 3 using 10 ^9^ tightly synchronized wild-type *P. falciparum* (strain 3D7) parasites harvested at 12 hours post-infection, and 75 µL protein G Dynabeads (Invitrogen 10004D) conjugated to 3 µg α-HA antibody (Abcam ab9110). fastq-dump.2 was used to download data generated previously from the NCBI Sequencing Read Archive ^16^ with the following accession numbers: control dCas9 input: SRR8802083 ^3^; control dCas9 IP: SRR8802084 ^3^; *var* dCas9 input: SRR8802087 ^3^; *var* dCas9 IP: SRR8802088 ^3^; α-GFP input: SRR16021005 ^4^; α-GFP IP: SRR16021003 ^4^; AP2-HC input: SRR12281322 ^5^; AP2-HC IP: SRR12281321 ^5^; HP1 IP: SRR12281320 ^5^; AP2-G input: SRR7903647 ^6^.

### Read mapping and filtering

Illumina sequencing adapters were trimmed from raw fastq files using trimmomatic (version 0.39) ^17^, removing poor-quality bases at both read ends (Phred score ≤ 20) and applying a 4 bp sliding-window trimming (option SLIDINGWINDOW:4:20). Only reads ≥ 50 nucleotides and proper read pairs (in the case of paired-end libraries) were retained. Trimmed reads were mapped to the *P. falciparum* genome ^7^ downloaded from plasmoDB.org (version v55) ^18^ with bowtie2 ^11^ using options --end-to-end and –sensitive. With these settings, bowtie2 reports only one alignment per read that can be further filtered for ‘uniqueness’ by its MAPQ value. For paired-end reads, the additional options --no-mixed and --no-discordant were used. PCR duplicates were filtered from the raw alignments with samtools ^19^ ‘fixmate’ and ‘markdup’ (with option -r). samtools ‘view’ was used to filter high quality ( *i.e.* more unique) sequencing alignments (MAPQ ≥ 30, option -q 30). For the MAPQ distribution ( Figure 2a), read alignments were processed using the same steps without the final MAPQ filtering step. Significant peaks in the α-HA ChIP-seq experiment were identified with macs2 ^20^ ‘callpeak’ using default settings and options --no-model and --extsize 150.

### Genome coverage calculation

BED files for eu- and heterochromatic regions for downstream analysis were generated using the HP1 ChIP-seq data generated in 21, with heterochromatic regions being those featuring enrichment of HP1 and euchromatic regions encompassing all remaining regions of the genome. Genic regions (coding sequences, CDS) were extracted from the *P. falciparum* genome annotation file (plasmoDB GFF, version 55). Intergenic regions were computed using the *P. falciparum* genome annotation file and bedtools ‘complement’ ^22^. Coverage plots of ChIP/input fold enrichments ( Figure 1a,b) were generated using deeptool’s bamCompare ^23^ by calculating the fold-enrichment between the IP and Input sample in 10 bp bins (option --bs 10) with options --operation ‘ratio’ and --normalizeUsing CPM. For the metagene plot of input samples, genome coverage was calculated using deeptool’s bamCoverage in bin sizes of 1000 bp (option --bs 1000) and normalized to counts per million (option --normalizeUsing CPM). The metagene was calculated using deeptool’s computeMatrix by scaling each euchromatic, central chromosome region in the 14 nuclear chromosomes to the same length and defining the flanking 80 kilobases as subtelomeric, heterochromatic regions. The graph was plotted using deeptool’s plotProfile with default settings.

Average genome coverages of hetero- and euchromatic regions and genes ( Figure 1c,e, Figure 2c,f) were calculated using mosdepth ^24^ with default settings. For between-sample comparisons, the total number of alignments were calculated using samtools flagstat and the average coverage per region was normalized to one million mapped reads (reads per million, RPM). Boxplots were generated in R using package ggplot2 ^25^. The GC-content analysis for the genome and the α-HA input sample was computed using ‘CollectGcBiasMetrics’ from the picard package.

### Mapping approach comparisons

To report all possible alignments of a given read, the input sample of the α-HA ChIP-seq experiment was mapped using bowtie2 as described above and with option -a and without further MAPQ filtering. Coverage plots ( Figure 2d) were generated using deeptool’s bamCoverage with option --bs 10, --normalizeUsing CPM. For the comparison with the MAPQ-filtered alignments, average genome coverage in the different genomic regions and genes was calculated using mosdepth ^24^ and normalized to reads per million (calculated using samtools flagstat from the quality filtered alignment file). All genome coverage tracks and read alignments ( Figure 1a, b and Figure 2d,e) were visualized using the Integrative Genomics Viewer (version 2.12.3) ^26^.

## Data availability

### Underlying data

NCBI BioProject: Systematic biases in genome-wide analyses of Plasmodium falciparum, https://identifiers.org/ncbiprotein:PRJNA832605

This project contains the sequencing data generated in this study.
