# Supplementary material for: Chromatin structure can introduce systematic biases in genome-wide analyses of Plasmodium falciparum
Source: Open Res Eur. 2022 Sep 15;2:75. Originally published 2022 Jun 10. [Version 2] doi: 10.12688/openreseurope.14836.2 (PMC10445928; doi:10.12688/openreseurope.14836.2)
Supplement: Peer review history

# Peer review: Reviewer response for version 2

**DOI:** 10.21956/openreseurope.16303.r30102

**Published:** 2022-09-23

**Authors:** Elena Gómez-Díaz, Diana C. López-Farfán

**Affiliations:** 1 Instituto de Parasitología y Biomedicina López-Neyra (IPBLN), Consejo Superior de Investigaciones Científicas, Granada, Spain

The authors have successfully addressed my previous concerns.

Is the study design appropriate and does the work have academic merit?

Partly

Is the work clearly and accurately presented and does it cite the current literature?

Yes

If applicable, is the statistical analysis and its interpretation appropriate?

Yes

Are all the source data underlying the results available to ensure full reproducibility?

Yes

Are the conclusions drawn adequately supported by the results?

Yes

Are sufficient details of methods and analysis provided to allow replication by others?

Yes

Reviewer Expertise:

Epigenomics, Malaria, Adaptation

We confirm that we have read this submission and believe that we have an appropriate level of expertise to confirm that it is of an acceptable scientific standard.

# Peer review: Reviewer response for version 1

**DOI:** 10.21956/openreseurope.16025.r29565

**Published:** 2022-07-25

**Authors:** Elena Gómez-Díaz, Diana C. López-Farfán

**Affiliations:** 1 Instituto de Parasitología y Biomedicina López-Neyra (IPBLN), Consejo Superior de Investigaciones Científicas, Granada, Spain

The manuscript by Sebastian Baumgarten *et al*. examines the impact of chromatin structure on ChIP-seq analysis in the malaria parasite Plasmodium falciparum. Authors evaluate how its unique genomic features impose strong bias and can lead to misleading results.

 The article is clearly written, well reasoned and correct in the claims and suggestions. In particular, the main claim of this work is that heterochromatic regions appear depleted in ChIP input controls because of technical and experimental issues and this can result in a false enrichment of heterochromatic regions in the tested sample. The authors present experimental evidence of the low sequencing read coverage of heterochromatic regions compared with euchromatic regions in several ChIP input samples, and how this bias affects normalisation of telomeric and sub-telomeric regions, which are important in P. falciparum virulence and pathogenesis. In the discussion they then make recommendations to improve the Chromatin IP experiments design and analysis.

 Although the claim is important, it is unclear which new insight the article and the reanalysis brings in terms of what factors are responsible for the bias, or the mitigation strategies that should be considered to eliminate that bias. Most recommendations are already covered in the ENCODE practical guidelines for the Analysis of ChIP-seq Data, for example, the use of an inespecific antibody (i.e. IgG) in addition to the input control (see https://www.ncbi.nlm.nih.gov/pmc/articles/PMC3431496/, https://pubmed.ncbi.nlm.nih.gov/24244136/ ). ^1^^,^^2^ The features that are responsible for the bias are also known in the malaria field: sequence composition (AT richness), complexity (sequence similarity of copy number genes), and chromatin structure. In fact, the original papers of the external ChIP-seq data referenced already apply most recommendations in terms of read mapping and quality control (for example see https://academic.oup.com/nar/article/50/6/3413/6548410). ^3^ 

 Perhaps it would be interesting to add further analysis on which telomeric/subtelomeric genes are more impacted or which life-cycle stages can be more affected. Also it would be great to combine ChIP-seq data with data measuring chromatin accessibility (for example ATAC-seq or MNAse-seq) to demonstrate how closed vs open regions are more or less biased in control vs test samples.

 Besides, we would suggest the authors to make the recommendations in terms of design, experiments and analysis more accessible, like a guideline or protocol for non-experts.

 Finally, the article would benefit from a more broad focus and perspective. For example, many of these problems raised also affect other NGS data types which are also impacted by chromatin structure and the intrinsic genomic characteristics of Pf. For example, in the case of ATAC-seq data coding regions which are GC rich compared with AT rich intergenic regions, are overrepresented due to PCR-amplification preference of GC-rich regions. In that case, it is important to include a transposed naked DNA as a control to correct the test data because of that bias in sequence composition. All mapping considerations that affect ChIP-seq data also affect ATAC-seq data for example. In that sense, why not expand recommendations to other types of NGS data like the ATAC-seq for which there is such a well-established ENCODE manual?

 Another area in which the recommendations made are very important is in CRISPR-Cas genomic experiments, very impacted by chromatin structure.

 Going even further, in the light of single-cell technologies I would suggest expanding on how these problems will impact single-cell ChIP-data.

 Other comments:

- Despite the fold enrichment of negative control ChIPs is lower/negligible to the specific ChIP enrichment, the peak calling analysis of control anti-HA ChIP for example, identified a lot of peaks that were significantly overrepresented within heterochromatic regions, this seems to indicate a problem with the normalization and peak calling analysis. Do authors recommend any bioinformatic analysis that can correct this false signal?
- To improve the figure legend 1, it is not fully self-explicative, you have to look in the text to fully understand it. E.g. In 1c, it is not stated that the input data is from six different ChIP-seq input libraries. In 1e, control is data from different controls ChIP together.
- The authors show that the underrepresentation of heterochromatic regions in the analyzed P. falciparum ChIP-seq input samples (Fig.1c), was not seen in the immunoprecipitated DNA from non-specific control antibodies (Fig. 1e, control), and said this leads to fake “enrichment” of heterochromatic regions when immunoprecipitated DNA is normalized to corresponding input DNA (Fig. 1a). However, it is not discussed why the genome coverage (RMP) of ChIP with control antibodies is not similar to the input chromatin. Are the euchromatin fragments lost during the ChIP process or is there a fraction heterochromatin fragments binding not specifically to the control antibodies, if the last is true, one possible solution that they did not comment on could be normalizing the ChIP experiment with the control non-specific ChIP instead of the input sample. The authors do comment on why the heterochromatin fragments could be underrepresented in the input sample (Fig 1c), e.g fragments of heterochromatic regions tend to be longer than those from euchromatic regions and are not sequenced as efficiently and/or are lost when the sonicated chromatin is cleared by centrifugation, but they don’t comment why the heterochromatin fragments are not underrepresented in the control IPs (Fig 1e. control)

Is the study design appropriate and does the work have academic merit?

Partly

Is the work clearly and accurately presented and does it cite the current literature?

Yes

If applicable, is the statistical analysis and its interpretation appropriate?

Yes

Are all the source data underlying the results available to ensure full reproducibility?

Yes

Are the conclusions drawn adequately supported by the results?

Yes

Are sufficient details of methods and analysis provided to allow replication by others?

Yes

Reviewer Expertise:

Epigenomics, Malaria, Adaptation

We confirm that we have read this submission and believe that we have an appropriate level of expertise to confirm that it is of an acceptable scientific standard, however we have significant reservations, as outlined above.

## References

[1] : ChIP-seq guidelines and practices of the ENCODE and modENCODE consortia. *Genome Res* .2012;22(9) : 10.1101/gr.136184.111 1813-31 10.1101/gr.136184.111 22955991PMC3431496

[2] : Practical guidelines for the comprehensive analysis of ChIP-seq data. *PLoS Comput Biol* .2013;9(11) : 10.1371/journal.pcbi.1003326 e1003326 10.1371/journal.pcbi.1003326 24244136PMC3828144

[3] : Genome-wide landscape of ApiAP2 transcription factors reveals a heterochromatin-associated regulatory network duringPlasmodium falciparum blood-stage development. *Nucleic Acids Research* .2022;50(6) : 10.1093/nar/gkac176 3413-3431 10.1093/nar/gkac176 PMC898953835288749

# Response

**Published:** 2022-08-28

**Authors:** Sebastian Baumgarten

**Affiliations:** Pasteur Institute, Paris, France

*The manuscript by Sebastian Baumgarten et al. examines the impact of chromatin structure on ChIP-seq analysis in the malaria parasite Plasmodium falciparum. Authors evaluate how its unique genomic features impose strong bias and can lead to misleading results.   The article is clearly written, well reasoned and correct in the claims and suggestions. In particular, the main claim of this work is that heterochromatic regions appear depleted in ChIP input controls because of technical and experimental issues and this can result in a false enrichment of heterochromatic regions in the tested sample. The authors present experimental evidence of the low sequencing read coverage of heterochromatic regions compared with euchromatic regions in several ChIP input samples, and how this bias affects normalisation of telomeric and sub-telomeric regions, which are important in P. falciparum virulence and pathogenesis. In the discussion they then make recommendations to improve the Chromatin IP experiments design and analysis.*

*Although the claim is important, it is unclear which new insight the article and the reanalysis brings in terms of what factors are responsible for the bias, or the mitigation strategies that should be considered to eliminate that bias. Most recommendations are already covered in the ENCODE practical guidelines for the Analysis of ChIP-seq Data, for example, the use of an inespecific antibody (i.e. IgG) in addition to the input control (see https://www.ncbi.nlm.nih.gov/pmc/articles/PMC3431496/, https://pubmed.ncbi.nlm.nih.gov/24244136/). The features that are responsible for the bias are also known in the malaria field: sequence composition (AT richness), complexity (sequence similarity of copy number genes), and chromatin structure. In fact, the original papers of the external ChIP-seq data referenced already apply most recommendations in terms of read mapping and quality control (for example see https://academic.oup.com/nar/article/50/6/3413/6548410).*

**Response: **We agree that the central ENCODE ChIP guidelines are clear and identical for any kind of species investigated (as stated in the introduction). However, as there have been so few chromatin/epigenetics studies in the Plasmodium field relative to model eukaryotes, we feel the standards have not been set as stringently. What we wanted to make clear and share with the community is that even when one follows these guidelines and performs the most stringent analysis possible, one can observe seemingly “specific” heterochromatic enrichment even in negative control samples. While some groups and researchers in the field might know about the particularities of the *P. falciparum* genome, the effects such features can have on genome-wide analysis has not been described in a systematic fashion yet. Our intention with this manuscript was not to provide new insight into the specific reasons of how and why this might happen (which is covered elsewhere already ^1,2^), but to make these specific biases in the *P. falciparum *genome known and clear to those who are new to chromatin biology in *P. falciparum* and/or do not have prior experience with such data but might be involved in collaborations or act as reviewers.  

*Perhaps it would be interesting to add further analysis on which telomeric/subtelomeric genes are more impacted or which life-cycle stages can be more affected. Also it would be great to combine ChIP-seq data with data measuring chromatin accessibility (for example ATAC-seq or MNAse-seq) to demonstrate how closed vs open regions are more or less biased in control vs test samples. * 

**Response:** In Figure 2e and 2f, we show how *var* genes that share high sequence similarity are particularly affected by different mapping approaches and have extended the text of these findings to other multigene families in the discussion. Because subtelomeric heterochromatin is dynamic between asexual and mosquito stages of the parasite, the biases in regard to sequencing coverage of input ChIP samples are likely also different between these life-cycle stages as now stated in the discussion). It is likely that closed chromatin is more refractory to sonication as reported elsewhere, which will lead to lower coverage across these regions and additional biases. ^2^ Notably though, it is known that intergenic regions are the most dynamic in terms of chromatin accessibility during the asexual replication of the parasite. ^3^ As shown in Figure 2a, these regions also feature substantially decreased mappability due to lower GC-content and sequence diversity. Therefore, intergenic regions are already biased against in terms of genome coverage (Fig. 2b) due to the intrinsic AT-richness, irrespective of chromatin accessibility. To our knowledge, there has not been a publication that includes an ATAC-seq and ChIP-seq dataset from the same parasite cell lines and time points. Thus, a direct and stringent comparison of biases in ChIP-seq samples due to open vs. closed chromatin states is not possible at this point. However, DNA sequence-based biases introduced with the Tn5 enzyme used for ATAC-seq have been analyzed and addressed in 3.  

*Besides, we would suggest the authors to make the recommendations in terms of design, experiments and analysis more accessible, like a guideline or protocol for non-experts.*  

**Response: **We provide fairly straightforward recommendations (often based on the ENCODE guidelines) with regard to experimental design and controls in the discussion. However, we also feel that it is very important for non-experts wanting to enter the field of epigenetics/chromatin/RNA biology in *P. falciparum* to work closely with experts and a skilled bioinformatician who have tested and established protocols which are difficult to convey with the necessary detail within the constraints of a manuscript. However, in an attempt to make the analysis description more accessible, we have added information on the performance of two other read mappers in the discussion section.  

*Finally, the article would benefit from a more broad focus and perspective. For example, many of these problems raised also affect other NGS data types which are also impacted by chromatin structure and the intrinsic genomic characteristics of Pf. For example, in the case of ATAC-seq data coding regions which are GC rich compared with AT rich intergenic regions, are overrepresented due to PCR-amplification preference of GC-rich regions. In that case, it is important to include a transposed naked DNA as a control to correct the test data because of that bias in sequence composition. All mapping considerations that affect ChIP-seq data also affect ATAC-seq data for example. In that sense, why not expand recommendations to other types of NGS data like the ATAC-seq for which there is such a well-established ENCODE manual?*  

**Response: **We agree that all biases that can arise during the analysis of sequencing data due to the intrinsic features of the *P. falciparum *genome (e.g. multi read-mappers, lower mapping efficiencies in AT-rich regions) likely affect different genome-wide analyses in a similar fashion, and we have added a paragraph in this regard to the updated version of the manuscript. Since we do not have the same extensive experience with ATAC-seq experiments as we do with ChIP-seq, however, we want to refrain from making recommendations for other researchers on how to perform such experiments. Moreover, we feel that the ATAC-seq publications from Toenhake et al. and Ruiz et al. have addressed these sequence-based biases adequately and make recommendations within the manuscript. ^3, 4 ^While RNA-seq, ATAC-seq, ChIP-seq, etc. will all suffer from DNA sequence-based biases due to library preparation, NGS, and analyses, ChIP-seq involves more experimental steps that introduce potential biases such as antibody specificity, crosslinking, and sonication. This, and the fact that we have more experience with and many more datasets for ChIP-seq is why we focus on this specific experiment type in our manuscript.  

*Another area in which the recommendations made are very important is in CRISPR-Cas genomic experiments, very impacted by chromatin structure.*

**Response: **This is very true, but is already well described and discussed elsewhere. ^5,6^  

*Going even further, in the light of single-cell technologies I would suggest expanding on how these problems will impact single-cell ChIP-data.*

**Response: **Since single-cell ChIP-seq relies on enzymatic (i.e. MNase) chromatin fragmentation that is also susceptible to chromatin structure, it is likely that similar intrinsic biases exist with this method. However, we (and nobody else in the *P. falciparum* field to our knowledge) have not tested this in the lab and therefore do not have the experimental evidence to make a statement in this regard.  

*Despite the fold enrichment of negative control ChIPs is lower/negligible to the specific ChIP enrichment, the peak calling analysis of control anti-HA ChIP for example, identified a lot of peaks that were significantly overrepresented within heterochromatic regions, this seems to indicate a problem with the normalization and peak calling analysis. Do authors recommend any bioinformatic analysis that can correct this false signal?*

**Response: **The peak calling analysis was performing as expected, because it does detect differences in coverage and read clustering between the IP and input sample after integrated background normalization. We included this analysis to highlight the fact that even when following a stringent mapping and filtering approach, one cannot rely on *p-*values alone, and that the observation described in this manuscript is not an issue that can primarily and exclusively be solved *in silico. *To ensure that the ChIP signal is truly of biological origin and to mitigate the risk of analyzing background enrichments, additional controls (e.g. IgG or negative control ChIP, verification of antibody specificity, ChIP followed by Western Blot) should be combined with orthogonal methods that help in cross-verifying ChIP signals. We emphasized this point in the discussion of the revised version of the manuscript. However, as shown in Fig 1a in the case where background enrichment co-occurs with specific enrichments, more stringent fold-enrichment and *p*-value cut-offs can exclude such peaks from downstream analysis.  

*To improve the figure legend 1, it is not fully self-explicative, you have to look in the text to fully understand it. E.g. In 1c, it is not stated that the input data is from six different ChIP-seq input libraries. In 1e, control is data from different controls ChIP together.*

**Response: **We have added additional explanation to this legend and also changed the color schemes in Figure 1d to discriminate data better from those shown in 1a.  

*The authors show that the underrepresentation of heterochromatic regions in the analyzed P. falciparum ChIP-seq input samples (Fig.1c), was not seen in the immunoprecipitated DNA from non-specific control antibodies (Fig. 1e, control), and said this leads to fake “enrichment” of heterochromatic regions when immunoprecipitated DNA is normalized to corresponding input DNA (Fig. 1a). However, it is not discussed why the genome coverage (RMP) of ChIP with control antibodies is not similar to the input chromatin. Are the euchromatin fragments lost during the ChIP process or is there a fraction heterochromatin fragments binding not specifically to the control antibodies, if the last is true, one possible solution that they did not comment on could be normalizing the ChIP experiment with the control non-specific ChIP instead of the input sample. The authors do comment on why the heterochromatin fragments could be underrepresented in the input sample (Fig 1c), e.g fragments of heterochromatic regions tend to be longer than those from euchromatic regions and are not sequenced as efficiently and/or are lost when the sonicated chromatin is cleared by centrifugation, but they don’t comment why the heterochromatin fragments are not underrepresented in the control IPs (Fig 1e. control)*

**Response: **The heterochromatin fraction of control IP samples analyzed in this manuscript is still lower than those of the respective euchromatin samples (Fig 1 e, ‘control’), although to a lesser degree as in the input sample and not significantly (p = 0.48). One possibility for why heterochromatin regions are less depleted in IP samples might be that control antibodies bind more often in an unspecific manner in protein-dense chromatin regions, and DNA within these regions therefore gets pulled-down more often, as was shown in other systems. ^7^ We added this point to the discussion of the updated manuscript version. Indeed, normalization to a control IP is another possibility to mitigate the effects of intrinsic biases, yet also comes with the introduction of other biases such as the differential pulldown of chromatin regions. ^7^ Control IP samples also feature lower sequence complexity and can therefore lead to uneven background coverage and overamplification, which can also lead to uneven background coverage. ^1^ We added a comment to the discussion of the updated version of the manuscript about how combining control IP and input DNA normalizations can help to additionally mitigate the risk of identifying false enrichments in ChIP-seq experiments of *Plasmodium*.    

**References**  

1. Landt, S. G. *et al.* ChIP-seq guidelines and practices of the ENCODE and modENCODE consortia. *Genome Res.* **22**, 1813–1831 (2012).
2. Teytelman, L. *et al.* Impact of chromatin structures on DNA processing for genomic analyses. *PLoS One* **4**, 1–11 (2009).
3. Toenhake, C. G. *et al.* Chromatin accessibility-based characterization of the gene regulatory network underlying *Plasmodium falciparum* blood-stage development. *Cell Host Microbe* **23**, 557-569.e9 (2018).
4. Ruiz, J. L. *et al.* Characterization of the accessible genome in the human malaria parasite *Plasmodium falciparum*. *Nucleic Acids Res.* **46**, 9414–9431 (2018).
5. Bryant, J. M., Baumgarten, S., Glover, L., Hutchinson, S. & Rachidi, N. CRISPR in Parasitology: Not Exactly Cut and Dried! *Trends Parasitol.* **35**, 409–422 (2019).
6. Lee, M. C. S., Lindner, S. E., Lopez-Rubio, J.-J. & Llinás, M. Cutting back malaria: CRISPR/Cas9 genome editing of *Plasmodium*. *Brief. Funct. Genomics* **18**, 281–289 (2019).
7. Xu, J. *et al.* To mock or not: a comprehensive comparison of mock IP and DNA input for ChIP-seq. *Nucleic Acids Res.* **49**, e17 (2021).

# Peer review: Reviewer response for version 1

**DOI:** 10.21956/openreseurope.16025.r29558

**Published:** 2022-06-22

**Authors:** Joana Santos

**Affiliations:** 1 Université Paris-Saclay, Paris, France

This is a well-written paper describing a rigorous study arising from an observation of the two authors when analysing ChIP-Seq data from a control experiment they conducted on the human malaria parasite *Plasmodium falciparum*. They re-analyse previously generated data plus generate new ChIP-Seq data and conclude that there is a strong bias against the mapping of heterochromatin regions in input samples from parasite chromatin. This is explained by the fact that the genome is very AT-rich, particularly in intergenic regions, and most heterochromatic regions are subtelomeric and highly similar. To counter-act this and prevent errors when analysing ChIP-Seq data, the authors suggest a series of aspects that should be taken into account when analysing data and the appropriate controls to include when doing ChIP-Seq experiments, in particular, and characterising parasite chromatin-associated proteins, in general.

 I think this article will improve the quality of the ChIP-Seq data generated by the malaria community and it will be seen as the "bible" of ChIP-Seq data analysis in the field.

 I have a few comments:

- In the introduction, the full name of ap2-g should be included to those unfamiliar with it and the way it is written it isn't clear that heterochromatic regions are rare in the parasite.
- This paper basically defines guidelines on how to better analyse ChIP-Seq data. To make these clear, I would add a table with them: input should be from same genomic prep, etc.

Is the study design appropriate and does the work have academic merit?

Yes

Is the work clearly and accurately presented and does it cite the current literature?

Yes

If applicable, is the statistical analysis and its interpretation appropriate?

Yes

Are all the source data underlying the results available to ensure full reproducibility?

Yes

Are the conclusions drawn adequately supported by the results?

Yes

Are sufficient details of methods and analysis provided to allow replication by others?

Yes

Reviewer Expertise:

parasitology, malaria, Toxoplasma, genomics

I confirm that I have read this submission and believe that I have an appropriate level of expertise to confirm that it is of an acceptable scientific standard.
